# Cincinnati Correspondence

**Published:** 1868-04-01

**Authors:** 


### CINCINNATI CORRESPONDENCE.

CINCINNATI, *March 5th*, 1868.

*Chicago Medical Journal:*

The dearth pertaining to matters of medical interest here has been somewhat relieved by the annual commencements of our medical schools. Again has Young Physic gone forth in his might of sheep-skin, text-book, and, perchance, amputating case, to recruit the medical army.

Y. P., like the ardent young soldier, will be sent to the *front*, to skirmish or do picket duty. May they imitate those progenitors who have never gone backward, but have ever stood to their colors!

The Cincinnati College of Medicine and Surgery — justly termed the "Mother of Professors" — had a graduating class of eleven. The valedictory was delivered by Prof. Vaughan, of the chair of chemistry.

To the Faculty of this school has been added, during the past season, the names of Professors Carroll and Young. The former comes forward as a teacher of Diseases of Women and Children, after half a century's experience as an intelligent and industrious practitioner. The latter, as Professor of Surgery, is entering a useful future. Educated in Medicine at Albany, under Dr. Swinburn, and during the whole period of the late war, as surgeon, a more than ordinary observer, he possesses more than usual ability.

On the 22nd of February, the Miami Medical College graduated thirty. Prof. Chapman delivered the customary address. During the evening, the graduated class and numerous professional friends were handsomely entertained by Prof. Mendenhall.

The exercise of the Ohio Medical College took place on the evening of March 2nd. Prof. P. S. Connor addressed the class, and was listened to with much interest. There were fifty-four graduates. A parting banquet was given by the

Faculty, at the St. Nicholas. The occasion was made partic- ticularly interesting by the presence of many of the friends of the school.

Last night, the Ohio Dental College graduated nine students.

The appearance of better times will doubtless add to the number of those who seek this point for medical education. The new hospital, which is being constructed on the Pavillion place, by the city, will be completed the coming summer; and as it will be of a first-class character, it will add to the attractiveness of our city.

There has been but little sickness here since the cholera disappeared (1866). Last fall and early winter, typhoid fever prevailed, principally among children and youths. This winter we have had an epidemic of measles, mild in character. Perhaps it would be well to include the *fashionable complaints* of typhoid fever and diphtheria, whose clinical history differs materially from those diseases enumerated in the nosology of anthors, under the same name.

The two complaints above referred to, have become endemic here, and are never fatal; in fact, many persons suffer from repeated attacks of both, during the season. ***

